# Emotional Experiences of Healthcare Workers in Biomedical Waste Management: A Qualitative Study From North India

**DOI:** 10.7759/cureus.113091

**Published:** 2026-07-21

**Authors:** Anurag Verma, Supriya, Parul, Udayakumar Rangasamy, Nitesh Verma, Ashutosh Mishra

**Affiliations:** 1 Community Medicine, Uttar Pradesh University of Medical Sciences, Saifai, IND

**Keywords:** biomedical waste management (bmwm), emotional experiences, healthcare workers, occupational health, qualitative research

## Abstract

Background

Biomedical waste management (BMWM) is an essential component of infection prevention and occupational safety within healthcare settings. While existing studies have largely focused on knowledge, compliance, and technical aspects of BMWM, limited evidence exists regarding the emotional experiences of healthcare workers (HCWs) involved in biomedical waste handling.

Objective

This study aimed to explore the emotional experiences of HCWs involved in BMWM and examine emotional responses across different BMWM workflow stages and HCW cadres.

Methods

An exploratory qualitative study was conducted among 42 HCWs, including staff nurses, resident doctors, laboratory technicians, and sanitation workers, at a tertiary care teaching hospital in North India between December 2025 and April 2026. Participants were selected using purposive sampling. Semi-structured in-depth interviews were conducted using an interview guide exploring emotional experiences associated with biomedical waste handling. Interviews were audio-recorded, transcribed verbatim, and analysed using reflexive thematic analysis.

Results

Four major themes emerged from the analysis: (1) fear and anxiety related to occupational exposure, (2) frustration and workplace challenges in BMWM, (3) adaptation, coping, and risk normalisation, and (4) professional responsibility and sense of contribution. Fear and anxiety were particularly prominent during sharps handling and accidental exposure situations, while frustration commonly stemmed from poor segregation practices, inadequate infrastructure, and inconsistent adherence to BMWM protocols. Participants described gradual adaptation to workplace risks over time, although some reported reduced vigilance with repeated exposure. Despite workplace challenges, many HCWs expressed a strong sense of professional responsibility and satisfaction associated with safe waste handling. Emotional experiences varied across workflow stages and HCW cadres.

Conclusion

HCWs involved in BMWM experience diverse emotional responses shaped by occupational risk, workplace environment, and professional responsibility. Existing BMWM interventions may benefit from integrating emotional preparedness, supportive supervision, and cadre-sensitive occupational health approaches alongside technical training to strengthen both worker wellbeing and safe waste management practices.

## Introduction

Biomedical waste management (BMWM) is an essential component of infection prevention and environmental safety within healthcare systems. According to the World Health Organization (WHO), nearly 15% of healthcare waste is hazardous and capable of causing infection, chemical injury, or environmental contamination if improperly handled [1]. Healthcare workers (HCWs), particularly those involved in waste segregation, sharps handling, transportation, and disposal, are routinely exposed to occupational risks including needlestick injuries, blood-borne infections, toxic exposure, and psychological stress [2]. In India, the challenge of BMWM is particularly important because of the large and growing healthcare system, increasing biomedical waste generation, and variability in institutional compliance with recommended waste handling protocols [3]. The Bio-Medical Waste Management Rules, 2016, established mandatory procedures for segregation, transport, treatment, and disposal; however, implementation gaps continue to be reported across healthcare settings [4].

Most research on BMWM among HCWs has focused on knowledge, attitudes, and practices (KAP), training status, and compliance with segregation protocols [5,6]. While such studies have improved the understanding of technical adherence, they provide limited insight into how HCWs emotionally experience BMWM in their day-to-day work. Existing evidence suggests that waste handlers and frontline HCWs frequently encounter fear of infection, anxiety following sharps exposure, fatigue, and workplace stress related to inadequate protective systems and institutional barriers [7]. However, these emotional experiences often remain poorly explored, especially in low- and middle-income countries where biomedical waste handling frequently occurs under constrained working conditions [8].

Emotions may substantially influence occupational behaviour and compliance with safe BMWM practices. Fear of exposure may increase vigilance, whereas repeated exposure without adverse outcomes may contribute to risk normalisation and reduced adherence to safety precautions. Similarly, frustration arising from inadequate infrastructure, staff shortages, or poor institutional enforcement may undermine motivation toward correct waste segregation and handling. Understanding emotional experiences across different stages of BMWM may therefore provide important insights into behavioural drivers that conventional KAP studies are unable to capture.

Despite increasing attention to biomedical waste safety, qualitative evidence exploring the emotional dimensions of BMWM among HCWs remains limited. Few studies have examined how healthcare personnel emotionally navigate different workflow stages of waste generation, segregation, sharps handling, transportation, and disposal. Therefore, the present study aimed to explore the emotional experiences of HCWs involved in BMWM in a tertiary care teaching hospital. Specifically, the study sought to identify emotions associated with different stages of the BMWM workflow, examine workplace factors shaping these emotional experiences, and understand the coping strategies adopted by HCWs.

## Materials and methods

Study design

An exploratory qualitative study was conducted using in-depth interviews to understand the emotional experiences of HCWs involved in BMWM. A qualitative approach was considered appropriate to explore subjective experiences, perceptions, and emotional responses associated with biomedical waste handling across different stages of routine workplace practice.

Study setting and duration

The study was conducted at the Uttar Pradesh University of Medical Sciences, a government tertiary care teaching hospital in Saifai, India, between December 2025 and April 2026. The hospital provides secondary and tertiary care services across multiple clinical departments and follows BMWM practices in accordance with the Bio-Medical Waste Management Rules, 2016 (as amended), issued by the Ministry of Environment, Forest and Climate Change, Government of India [4]. Biomedical waste generated at the facility is managed through colour-coded segregation, temporary storage, internal transportation, and disposal through an authorised common biomedical waste treatment facility.

Study participants and sampling

Purposive sampling was employed to recruit HCWs directly involved in BMWM activities. Participants were selected to ensure representation from four professional cadres: staff nurses, resident doctors, laboratory technicians, and sanitation/housekeeping workers. HCWs with at least six months of experience in BMWM or related activities were eligible for inclusion. Of the 56 HCWs approached, 48 met the eligibility criteria. Four eligible participants declined participation because of unwillingness or inability to participate, and two interviews were not completed (one due to an incomplete interview and one due to poor audio recording). The final analytical sample comprised 42 participants, including 12 staff nurses, 10 resident doctors, 10 laboratory technicians, and 10 sanitation/housekeeping workers. Recruitment continued until sufficient information power was achieved, with concurrent review of interview data indicating that no substantively new themes or insights were emerging across participant groups. The research team reviewed emerging codes and themes throughout data collection, and recruitment was discontinued when additional interviews primarily reinforced existing patterns rather than contributing new conceptual insights.

Data collection tool and procedure

Data were collected using a semi-structured in-depth interview guide developed after reviewing published literature on BMWM, occupational experiences of HCWs, and workplace safety. The interview guide was informed by the Bio-Medical Waste Management Rules, 2016 (as amended), and explored participants' experiences across key stages of the BMWM workflow, including waste generation, segregation, sharps handling, temporary storage, internal transportation, final disposal, accidental exposure situations, and end-of-shift reflections. The complete interview guide is provided in the Appendices.

In-depth interviews were conducted in a private setting within the hospital premises by trained investigators. Interviews were conducted in either Hindi or English according to participants' preferences and were audio-recorded after obtaining written informed consent. Field notes were maintained to document contextual observations and non-verbal responses. Each interview lasted approximately 20-30 minutes.

Data analysis

Audio recordings were transcribed verbatim. Interviews conducted in Hindi were translated into English by the first author, who is fluent in both languages. To preserve conceptual meaning and contextual nuances, translated transcripts were reviewed alongside the original recordings during analysis. Representative participant quotations included in the manuscript were translated as faithfully as possible while retaining their original intent and meaning.

Reflexive thematic analysis was conducted.The first author performed the initial coding manually without the use of qualitative data analysis software. Codes were generated inductively from the data and iteratively refined into broader themes through repeated review of the transcripts. Emerging codes and themes were discussed with the co-authors during regular analytical meetings to enhance interpretive rigour and ensure consistency. Differences in interpretation were resolved through discussion and consensus rather than statistical measures of agreement, consistent with a reflexive thematic analysis approach.

Data collection and analysis were conducted concurrently, allowing emerging patterns and themes to inform ongoing recruitment decisions until sufficient information power was achieved.

Following thematic analysis, emotional narratives were examined across key BMWM workflow stages to explore variation in emotional experiences during routine waste handling. Commonly described emotions, including fear, frustration, fatigue, pride, and relief, were mapped using a qualitative, participatory rural appraisal (PRA)-inspired approach to provide an interpretive visual representation of emotional salience and overlap across workflow stages.

Trustworthiness

Credibility of findings was enhanced through investigator triangulation, repeated review of transcripts, and discussion of emerging themes among the research team. Member validation was performed for a subset of participants to ensure consistency between participant experiences and interpretation of findings. Reflexive discussions were conducted during the analytical process to minimise interpretive bias.

Ethical considerations

Ethical approval was obtained from the Institutional Ethics Committee of the Uttar Pradesh University of Medical Sciences prior to the commencement of the study (approval number: 158/2025-26). Written informed consent was obtained from all participants prior to interviews, including consent for audio recording. Confidentiality and anonymity were maintained throughout the study, and participants were informed of their right to withdraw at any stage without consequences.

## Results

Participant characteristics

Table 1 summarises the sociodemographic and occupational characteristics of the study participants. The study included HCWs from four professional cadres with varying ages, work experience, training status, and occupational exposure histories, providing a diverse sample for exploring emotional experiences related to BMWM.

**Table 1:** Characteristics of the study participants (N=42) BMWM: biomedical waste management; SD: standard deviation

Characteristic	Staff nurses (n=12)	Resident doctors (n=10)	Laboratory technicians (n=10)	Sanitation workers (n=10)	Total (N=42)
Sex					
Male, n (%)	3 (25)	6 (60)	7 (70)	8 (80)	24 (57.1)
Female, n (%)	9 (75)	4 (40)	3 (30)	2 (20)	18 (42.9)
Mean age, years (SD)	31.4 (4.2)	27.8 (2.1)	34.6 (6.7)	38.2 (8.4)	32.7 (6.1)
Work experience					
<2 years, n (%)	2 (16.7)	5 (50)	1 (10)	1 (10)	9 (21.4)
2-5 years, n (%)	6 (50)	4 (40)	4 (40)	3 (30)	17 (40.5)
>5 years, n (%)	4 (33.3)	1 (10)	5 (50)	6 (60)	16 (38.1)
BMWM training in past year, n (%)	10 (83.3)	6 (60)	8 (80)	4 (40)	28 (66.7)
Prior accidental exposure/needlestick event, n (%)	4 (33.3)	3 (30)	2 (20)	5 (50)	14 (33.3)
Primary interview language: Hindi, n (%)	7 (58.3)	5 (50)	6 (60)	10 (100)	28 (66.7)

Thematic analysis

Thematic analysis identified four major themes reflecting participants' emotional experiences during BMWM. Table 2 provides an overview of these themes, their descriptions, and representative participant quotations.

**Table 2:** Summary of themes, description, and representative participant quotations BMWM: biomedical waste management; PPE: personal protective equipment

Theme	Description	Representative participant quotation	Cadre
Theme 1: Fear and Anxiety Related to Occupational Exposure	Fear related to sharps handling, accidental exposure, infection risk, and handling contaminated waste	"Whenever I am handling needles or sharp items, I become extra careful because even one mistake can create a big problem."	Staff nurse
		"After a needlestick injury, I remained worried for many days even though my reports were normal."	Resident doctor
		"Sometimes there is fear in the back of the mind, especially if waste is mixed or not disposed properly."	Laboratory technician
Theme 2: Frustration and Workplace Challenges in BMWM	Frustration due to poor segregation practices, mixed waste, inadequate PPE, and lack of institutional support	"Even if we segregate properly, sometimes others throw waste in the wrong bins, which becomes frustrating."	Resident doctor
		"Sometimes we feel that nobody notices the difficulty of this work."	Sanitation worker
		“During busy shifts, proper disposal becomes difficult if bins are already full.”	Staff nurse
Theme 3: Adaptation, Coping, and Risk Normalisation	Adjustment to repeated exposure, routine familiarity, and coping with workplace stress	"In the beginning I was very scared, but now I know how to manage things better."	Staff nurse
		"After working for many years, sometimes people become too casual because nothing serious has happened before."	Laboratory technician
		"You slowly get used to the work; otherwise, it becomes difficult to continue."	Sanitation worker
Theme 4: Professional Responsibility and Sense of Contribution	Feelings of responsibility, contribution to safety, and satisfaction from proper BMWM practices	"When waste is managed properly, I feel satisfied because it helps prevent infection for others."	Laboratory technician
		"If someone appreciates our work, it feels good because this work is also important for the hospital."	Sanitation worker
		"Proper disposal is also part of patient care."	Resident doctor

Theme 1: Fear and Anxiety Related to Occupational Exposure

Fear of occupational exposure emerged as one of the most frequently reported emotional experiences across all cadres. Participants commonly described feelings of caution, anxiety, and heightened alertness, particularly during sharps handling and accidental exposure situations. Nurses and laboratory technicians frequently expressed concern regarding needlestick injuries and blood-borne infections, while sanitation workers described a more generalised fear associated with direct waste contact.

Several participants reported becoming especially cautious during sharps disposal and handling of high-risk waste.

"Whenever I am handling needles or sharp items, I become extra careful because even one mistake can create a big problem." (Staff Nurse)

"Sometimes there is fear in the back of the mind, especially if waste is mixed or not disposed properly." (Laboratory Technician)

Participants with a prior accidental exposure experience often described persistent anxiety even after medical evaluation.

"After a needlestick injury, I remained worried for many days even though my reports were normal." (Resident Doctor)

Fear was reported to be most intense during sharps handling and accidental exposure stages of BMWM, whereas anxiety reduced after safe disposal or completion of the task.

Theme 2: Frustration and Workplace Challenges in BMWM

Frustration related to workplace conditions and institutional practices was another prominent theme. Participants frequently described dissatisfaction related to inadequate segregation practices, improper disposal by colleagues, shortage of protective equipment, and overfilled waste bins.

Resident doctors and nurses often expressed frustration when proper segregation practices were not followed consistently by coworkers, making safe waste handling difficult.

"Even if we segregate properly, sometimes others throw waste in the wrong bins, which becomes frustrating." (Resident Doctor)

Sanitation workers described challenges related to handling mixed waste and perceived lack of acknowledgement of their work.

"Sometimes we feel that nobody notices the difficulty of this work. We handle waste every day but people do not think about it." (Sanitation Worker)

Several participants linked frustration to infrastructure-related issues, including delayed collection, poor maintenance of storage areas, and inadequate availability of gloves or masks during busy periods.

Theme 3: Adaptation, Coping, and Risk Normalisation

Participants described adapting to BMWM-related stress over time through personal coping mechanisms and routine work practices. Experienced HCWs commonly reported becoming more comfortable with waste handling responsibilities after repeated exposure.

While some participants viewed adaptation positively as increased confidence and familiarity with procedures, others expressed concern that routine exposure occasionally led to reduced caution.

"In the beginning I was very scared, but now I know how to manage things better." (Staff Nurse)

"After working for many years, sometimes people become too casual because nothing serious has happened before." (Laboratory Technician)

Common coping strategies included peer support, increased attention during high-risk procedures, adherence to personal protective measures, and emotional detachment from routine waste-related tasks.

Fatigue related to repeated exposure and demanding work schedules was also commonly reported, particularly among sanitation workers and nurses.

Theme 4: Professional Responsibility and Sense of Contribution

Despite challenges, many participants expressed a sense of professional responsibility toward safe BMWM practices. Participants frequently linked proper waste handling to prevention of infection, worker safety, and protection of patients and the hospital environment.

Nurses and laboratory technicians particularly described satisfaction associated with proper segregation and disposal.

"When waste is managed properly, I feel satisfied because it helps prevent infection for others." (Laboratory Technician)

Some sanitation workers reported feeling motivated when their contribution was recognised by colleagues or supervisors.

"If someone appreciates our work, it feels good because this work is also important for the hospital." (Sanitation Worker)

Participants generally viewed BMWM as an important but often overlooked component of healthcare delivery.

Emotional experiences across BMWM workflow stages

**Figure 1:**
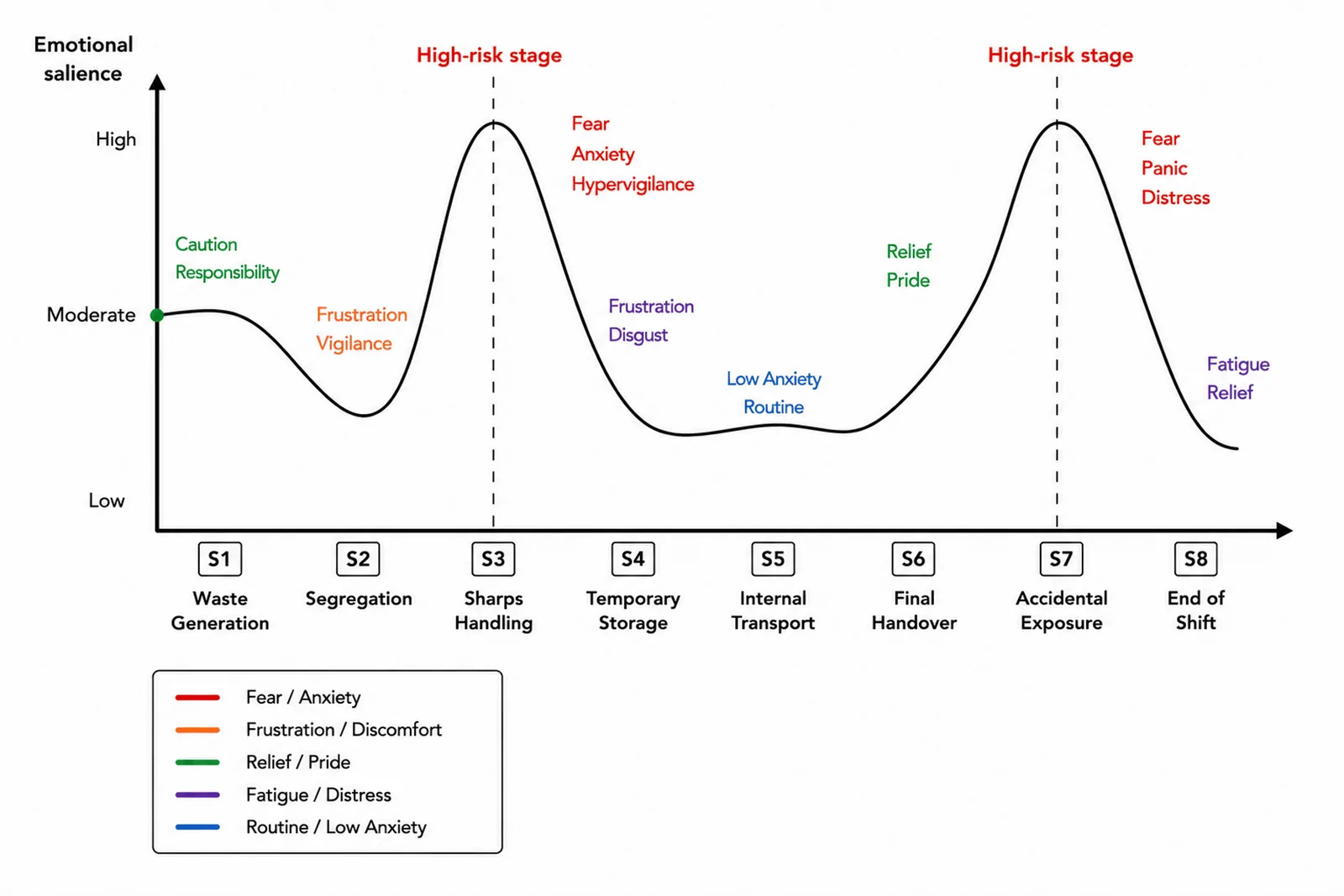
Visual mapping of emotional experiences during BMWM BMWM: biomedical waste management Conceptual visual mapping of emotional experiences across the BMWM workflow. This interpretive visualisation was developed from reflexive thematic analysis of participant narratives. The relative prominence of emotions represents qualitative interpretation rather than quantitative frequency or intensity and should not be interpreted as a statistical comparison. Fear and anxiety were most pronounced during sharps handling and accidental exposure, whereas frustration was commonly associated with waste segregation and temporary storage. Relief and professional pride were more frequently expressed following successful waste disposal, while fatigue was commonly reported at the end of the work shift. This figure was created using Adobe Illustrator (Adobe Inc.,  San Jose, California, United States).

To further illustrate how these emotional experiences varied across different stages of the BMWM workflow, a conceptual visual map was developed based on the thematic analysis. Figure 1 provides an integrated representation of the relative emotional salience reported by participants across key workflow stages.

Table 3 provides a stage-wise summary of the commonly reported emotional responses and their associated triggers across the BMWM workflow, complementing the conceptual visual representation shown in Figure 1.

**Table 3:** Common emotional experiences across BMWM workflow stages BMWM: biomedical waste management

BMWM workflow stage	Frequently reported emotional responses	Commonly reported trigger(s)
1. Waste generation at point of care	Caution, vigilance, responsibility	Handling contaminated materials during patient care
2. Waste segregation into colour-coded bins	Frustration, attentiveness, responsibility	Improper segregation practices by colleagues
3. Sharps and high-risk waste handling	Fear, anxiety, heightened alertness	Risk of needlestick injury or accidental exposure
4. Temporary storage and collection	Frustration, discomfort, disgust	Overfilled bins, delayed collection, unpleasant conditions
5. Internal transportation of waste	Routine, fatigue, emotional detachment	Repetitive handling and movement of waste
6. Final disposal/handover	Relief, satisfaction, task completion	Safe disposal and completion of assigned responsibility
7. Accidental exposure situations	Fear, stress, worry	Needlestick injury, contact with contaminated waste
8. End-of-shift reflections	Fatigue, relief, emotional exhaustion	Completion of work responsibilities

## Discussion

The present study explored the emotional experiences of HCWs involved in BMWM and identified variations in emotional responses across different workflow stages and HCW cadres. Four major themes emerged: fear and anxiety related to occupational exposure, frustration associated with workplace and institutional barriers, adaptation and coping through repeated exposure, and a sense of professional responsibility despite occupational challenges. Emotional responses were found to vary across BMWM stages, with fear being most prominent during sharps handling and accidental exposure, while relief and satisfaction were commonly experienced following safe disposal and task completion.

Fear and anxiety related to occupational exposure emerged as one of the most commonly reported emotional experiences among participants, particularly during sharps handling and accidental exposure situations. Participants frequently described persistent worry regarding infections following needlestick injuries and concerns regarding improper segregation of contaminated waste. These findings are consistent with literature highlighting the psychological burden associated with occupational exposure among HCWs. Hambridge et al. reported that sharps injuries may lead to prolonged psychological distress, including anxiety and fear, while psychological support following such incidents often remains inadequate [9]. Similarly, a study from Lao People's Democratic Republic (PDR) demonstrated significantly higher anxiety levels among HCWs who had experienced needlestick and sharp injuries, with fear persisting weeks after the incident despite medical reassurance [10]. Our findings align with these observations, as participants often described lingering apprehension even after medical evaluation and negative reports.

The emotional burden observed in the present study may also reflect the substantial occupational risk associated with healthcare waste handling in low-resource settings. Studies from India have reported high prevalence of needlestick injuries and occupational exposure among HCWs, particularly among nurses and resident doctors [11,12]. Although these studies primarily quantified occupational exposure and underreporting, rather than emotional experiences, they provide important contextual evidence supporting the legitimacy of fear expressed by participants in the current study. Likewise, Senthil et al. reported widespread biological exposure among HCWs in southern India, accompanied by psychological stressors and negative emotional experiences, suggesting that occupational risks may extend beyond physical hazards alone [13].

Frustration related to workplace barriers and institutional practices constituted another important finding. Participants frequently expressed dissatisfaction regarding poor waste segregation practices, inadequate personal protective equipment (PPE), delayed waste collection, and inconsistent adherence to BMWM protocols by coworkers. Similar operational barriers have been documented in BMWM literature. A recent study from eastern India identified poor segregation practices, inadequate infrastructure, and inconsistent compliance as major contributors to ineffective BMWM, although improvements were observed following training and systems strengthening interventions [6]. The frustration expressed by participants in the present study may therefore represent a practical response to institutional gaps that complicate safe waste handling practices.

Differences across worker cadres were also apparent. Sanitation workers frequently described feelings of invisibility and inadequate recognition despite regular exposure to biomedical waste, while resident doctors and nurses more commonly expressed frustration with poor compliance among colleagues. Previous studies have similarly highlighted disparities in knowledge and preparedness across healthcare cadres, particularly among sanitation staff [14,15]. For example, studies from Delhi and West Bengal reported poorer awareness regarding BMWM practices among sanitary workers compared with doctors and nurses, potentially increasing vulnerability to unsafe practices and occupational stress [14,15]. Findings from Uttar Pradesh further suggest that implementation of BMW guidelines remains inconsistent despite the presence of formal regulations, underscoring the importance of supportive institutional environments for effective BMWM [16].

Another important finding was the process of adaptation and coping reported by participants over time. Several HCWs described becoming more comfortable with biomedical waste handling following repeated exposure, whereas some participants also acknowledged reduced vigilance due to familiarity with workplace risks. These findings may reflect a process of occupational adaptation, wherein repeated exposure to hazards gradually modifies risk perception. A study on occupational risk perception among HCWs found that repeated exposure to biological hazards often shapes workers' understanding and emotional response to risk, with familiarity influencing vigilance and behaviour [17]. Similarly, a scoping review of occupational hazards among HCWs in low- and middle-income countries reported persistent exposure to biological and psychosocial hazards, often occurring within resource-constrained environments where safety systems remain suboptimal [18]. Such repeated exposure may contribute to gradual emotional adjustment and normalisation of occupational risk.

At the same time, participants in the present study described fatigue and emotional exhaustion associated with repetitive waste handling, particularly among sanitation workers and nursing staff. Such experiences may reflect the cumulative effects of repeated occupational exposure and demanding workplace conditions. The gradual acceptance of workplace hazards as "part of routine work," as observed among some participants, may represent a form of occupational risk normalisation. While adaptation may improve confidence and efficiency, reduced vigilance over time may also increase vulnerability to unsafe practices and accidental exposure.

Despite occupational challenges, many participants described a strong sense of responsibility toward safe BMWM practices and perceived waste handling as an important contributor to patient and workplace safety. Feelings of satisfaction and contribution were particularly evident after safe disposal or recognition of work efforts. Similar findings have been reported in qualitative healthcare literature examining meaning in work and professional identity. Lamothe and Guay observed that HCWs continued to derive meaning and professional fulfilment from their contribution to patient care despite workplace adversity [19]. Likewise, studies exploring nursing identity have reported that professional responsibility and commitment to patient protection often remain strong even in demanding workplace settings [20,21]. In the context of BMWM, such professional commitment may represent an important facilitator of sustained adherence to safe waste management practices.

The findings of this study have important practical implications. Existing BMWM interventions largely focus on technical training, colour coding, and regulatory compliance, with relatively limited attention to the emotional and psychosocial dimensions of waste handling. Incorporating emotional preparedness, psychological support following accidental exposure, cadre-sensitive training, and supportive supervision may strengthen both worker wellbeing and adherence to safe BMWM practices. Greater recognition of sanitation workers' contributions and improved institutional support systems may also reduce frustration and improve engagement with BMWM protocols.

This study has several limitations. As a qualitative study conducted in a single tertiary care teaching hospital, the findings are context-specific and may not be transferable to all healthcare settings. Purposive sampling may have introduced selection bias, although it enabled the inclusion of participants with diverse roles in BMWM. Interviews conducted in Hindi were translated into English for analysis, which may have resulted in subtle loss of linguistic nuance despite efforts to preserve conceptual meaning. Additionally, the conceptual visual mapping of emotional experiences represents an interpretive synthesis of qualitative findings and should not be considered a quantitative representation of emotional frequency or intensity.

The findings of this study have practical implications for strengthening BMWM programmes in healthcare settings. Interventions should extend beyond technical training to include emotional support, recognition of the contributions of HCWs involved in BMWM, and measures to reduce stigma associated with waste handling. Incorporating psychosocial well-being into infection prevention and BMWM policies may improve worker safety, job satisfaction, and adherence to recommended waste management practices.

## Conclusions

HCWs involved in BMWM experience a range of emotional responses shaped by occupational exposure, workplace conditions, and professional responsibilities. Fear and anxiety were particularly evident during sharps handling and accidental exposure situations, whereas frustration frequently arose from institutional barriers such as poor segregation practices and inadequate workplace support. Repeated exposure to biomedical waste contributed to adaptation and coping among many participants, although concerns regarding risk normalisation and reduced vigilance were also identified. Despite these challenges, HCWs continued to perceive BMWM as an important responsibility contributing to patient, worker, and environmental safety.

The findings highlight the need to move beyond technical compliance-focused BMWM training by incorporating emotional preparedness, psychological support following occupational exposure, and cadre-sensitive workplace interventions. Strengthening institutional support systems and recognising the contributions of sanitation workers and other frontline staff may improve both worker wellbeing and adherence to safe BMWM practices.

## Appendices

**Table 4:** Semi-structured in-depth interview guide BMWM: biomedical waste management; PPE: personal protective equipment The interview guide was semi-structured. Additional probing questions were used, where appropriate, to encourage participants to elaborate on their experiences.

Phase/domain	Interview questions	Probes (if applicable)
Introduction and consent	Introduction to the study, confidentiality, voluntary participation, and permission for audio recording	Study explained; opportunity for questions; written informed consent; permission for audio recording
Rapport building	1. Could you briefly introduce yourself and describe your role in this hospital?	-
	2. What are your routine responsibilities related to BMWM?	
Domain 1: experiences during BMWM	3. Can you describe your usual experience while handling biomedical waste during your daily work?	• What types of biomedical waste do you usually handle?
		• Which BMWM activities are you most involved in?
Domain 2: experiences across different workflow stages	4. Can you describe your experiences during the following stages of BMWM: waste generation, waste segregation, sharps/high-risk waste handling, temporary storage and collection, internal transportation, final disposal or handover, accidental exposure situations (if experienced), and at the end of your work shift?	• Which stage do you find most challenging?
		• Can you describe any memorable incident?
		• How do these experiences differ across stages?
Domain 3: workplace factors	5. What workplace situations or conditions influence your experiences while handling biomedical waste?	• Availability of PPE
		• Waste segregation practices
		• Infrastructure and waste collection
		• Workload
		• Colleague or supervisory support
Domain 4: changes over time	6. Have your experiences related to biomedical waste handling changed since you first started this work?	• Changes with increasing experience
		• Confidence or familiarity
		• Changes in your approach over time
Domain 5: professional meaning	7. What does safe BMWM mean to you?	• Patient safety
		• Protection of healthcare workers
		• Environmental safety
		• Personal responsibility
Domain 6: recommendations	8. What changes do you think would improve the experiences of healthcare workers involved in BMWM?	• Training
		• Workplace support
		• Infrastructure
		• Safety measures
		• Institutional policies
Closing	9. Is there anything else you would like to share about your experiences with BMWM that we have not discussed?	Thank the participant for their time and contribution
